# Supplementary material for: Impact of transport modality on time to endovascular thrombectomy: A population-based registry study in rural Sweden
Source: Scand J Trauma Resusc Emerg Med. 2026 Feb 26;34:52. doi: 10.1186/s13049-026-01587-4 (PMC12954892; doi:10.1186/s13049-026-01587-4)
Supplement: Supplementary file 2 — Supplementary Material 2. [file 13049_2026_1587_MOESM2_ESM.docx]

Supplement 1

**Destination hospital matrix for Strokepatients in VGR**

(HEMS Specific)

**mNIHSS 2-5**

Is it <4.5h from symptom onset?

YES: Proceed to the nearest PSC.

NO: Is time saved with HEMS?

YES: Proceed to the nearest PSC.

NO: No HEMS activation.

**mNIHSS ≥6**

Scenario 1: <4.5h from symptom onset

Sub-scenario A: NOTE: Transport time to CSC prolongs the start of Thrombolysis by >30 min

Is time saved with HEMS to the nearest emergency hospital?

YES: HEMS activiation

NO: No HEMS activiation

Sub-scenario B: NOTE: Transport time to CSC does not prolong the start of Thrombolysis by >30 min

Is time saved with HEMS?

YES: Contact CSC. This leads to HEMS activation.

NO: No HEMS activation.

Scenario 2: >4.5h - <9h from symptom onset to arrival at CSC. HEMS provides perceived time benefit.

YES: Contact CSC. This leads to HEMS activation.

NO: Proceed to Scenario 3.

Scenario 3: >9h - <24h

YES: Perceived time benefit with HEMS to the nearest PSC?

YES: HEMS activation.

NO: No HEMS activation

**Prehospital Treatment Guidelines**

In case of suspected stroke symptoms with unclear time of onset or onset within 24 hours, an ambulance must be dispatched with **Priority 1**.

The ambulance must have a checklist for the identification and documentation of:

- Symptom profile suggestive of stroke.
- Severity of stroke according to the **(m)NIHSS** (modified version of the National Institutes of Health Stroke Scale)
- Indications and contraindications for reperfusion treatment.

**Indications and Contraindications for Stroke Alert**

**Indications**

- Age ≥16 years. And a) or b) or c):

a) (m)NIHSS ≥1 and symptom onset time such that thrombolysis can be initiated <4.5 hours.

b) (m)NIHSS ≥3 and symptom onset 4.5–24 hours. Thrombectomy must be able to be initiated there <24 hours after secondary transport to SU/Sahlgrenska. In case of unclear time of onset without signs that the onset occurred >24 hours, the patient is managed as if within 24 hours.

c) (m)NIHSS <3 and symptom onset 4.5–24 hours. If symptoms include aphasia, neglect, or visual field loss, the responsible stroke attending physician at the receiving hospital can be contacted for discussion as to whether the patient may still be eligible for a stroke alert.

**Contraindications**

- Comorbidity that makes the benefit of reperfusion treatment unlikely. Contact the responsible stroke attending physician at the receiving hospital for assessment.
- Decreased level of consciousness is not an impediment to reperfusion treatment if other causes of decreased consciousness have been evaluated and the primary suspicion of stroke remains. However, a patient with decreased consciousness should initially be assessed and, if necessary, stabilized in the emergency department before further management according to the stroke alert if stroke suspicion persists.

**Actions:**

- To enable rapid reperfusion treatment, Priority 1 for ambulance transports should be applied throughout the stroke care chain concerning reperfusion.
- Prioritize departure (rapid transport).
- Ascertain and note the time of symptom onset.
- Alert the receiving healthcare unit.
- Intravenous access (green peripheral IV catheter), preferably two on the left side. If transport to SU is sought, aim for three on the left side; if not possible, two on the left (thrombectomy) and one on the right (thrombolysis) side. This action must not delay departure.
- Monitor vital signs such as oxygen saturation, respiratory rate, pulse, blood pressure, temperature, blood glucose, and level of consciousness.
- If blood glucose < 3, treat with IV glucose.
- If SpO2 < 95%, administer 1–3 L of oxygen via nasal cannula.
- In case of nausea, administer ondansetron 4 mg IV.

**Choice of Receiving Hospital**

Basic rule: All patients where reperfusion treatment can be initiated within 24 hours are transported to the nearest acute care hospital (either for thrombolysis and/or for evaluation for thrombectomy).

**Exceptions:**

1. Direct transport to SU/Sahlgrenska (CT lab at The Center for Imaging and Intervention, floor 0):

o Patients outside SU's catchment area but <45 minutes transport time to SU/Sahlgrenska and with (m)NIHSS ≥6 who can reach SU/Sahlgrenska <6 hours from symptom onset for thrombectomy and after consultation with the thrombolysis attending physician at SU/Sahlgrenska (031-342 87 14). Patients >45 minutes ambulance transport time to SU/Sahlgrenska and with (m)NIHSS ≥6, who cannot reach the nearest acute care hospital for thrombolysis (<4.5 hours from symptom onset), but can reach SU/Sahlgrenska <6 hours from symptom onset for thrombectomy and after consultation with the thrombolysis attending physician at SU/Sahlgrenska (031-342 87 14).

o For patients in ambulances equipped with video links outside SU's catchment area, who can reach SU/Sahlgrenska <6 hours from symptom onset, with (m)NIHSS ≥6 and after consultation with the thrombolysis attending physician (031-342 87 14) weekdays 09:00-16:00; all other times including weekends, the regional reperfusion attending physician at SU (via SU switchboard 031-342 70 00).

1. Total time for transport to the first hospital and secondary transport to SU/Sahlgrenska yields a clear time saving if choosing a hospital other than the nearest hospital (mainly concerns the western parts of SkaS's catchment area):

o Patients with (m)NIHSS ≥6 within 24 hours after symptom onset with a transport time no more than 15 minutes longer to another hospital than to the nearest hospital are transported to the other hospital.

**Await Decision on Secondary Transport to SU/Sahlgrenska:**

- When dropping off patients with (m)NIHSS >6 at an acute care hospital (not SU/Sahlgrenska), the ambulance should wait for 20 minutes for a decision (yes/no) regarding the need for secondary transport for thrombectomy (i.e., until after assessment with CT angiography and possibly CT perfusion). This presupposes that the patient has been handed over at the CT lab and that any thrombolysis can be initiated there before the secondary transport starts. Exception if the emergency call center needs the ambulance resource for a life-threatening mission or near a shift change, where another ambulance can take over any secondary transport.

**Secondary Transport to SU/Sahlgrenska for Patients Judged to Require Thrombectomy:**

- If possible, ensure the patient has three intravenous lines (preferably on the left side). If not possible, two on the left (thrombectomy) and one on the right (thrombolysis) side. This action must not delay departure.
- If thrombolysis (inj. Tenecteplase has been given or inf. Alteplase is running):

o Check level of consciousness (RLS), mNIHSS, pulse, and blood pressure every 15 minutes.

o If blood pressure is above 185/110, contact a physician at the departure hospital for an order for antihypertensive medication. If there is a risk of blood pressure requiring treatment during transport, appropriate medication should be sent with the ambulance.

o In case of suspicion of complications (see under the heading "Complications during Thrombolysis"), contact a physician at the departure hospital. Any Alteplase infusion should be stopped.

- Transport the patient directly to Intervention 1/Neurointervention, floor 2/3 SU/S. It is recommended to use the ambulance entrance Bruna Stråket 8, SU/S.
- Alert the thrombolysis attending physician at SU/Sahlgrenska 15 minutes before arrival. Report any changes in the patient's status such as (m)NIHSS, level of consciousness (RLS), pulse, and blood pressure.
- Call the emergency orderly/porter or SU switchboard 15–20 minutes before arrival for a meeting and help with locked elevator doors, etc.
- In the ambulance before arrival at the neurointervention lab, prepare the patient for rapid management in the lab; remove the patient's shoes and prepare for removal of pants (unbutton, etc.).
- Give a brief report on the changed status inside the neurointervention lab. The report should include the blood pressure the patient had at the first check, as this is important during the thrombectomy.

**Return Transport of Patients from SU/Sahlgrenska to the Region's Other Hospitals** after acute radiological evaluation or treatment. This applies to patients who are not part of SU/Sahlgrenska's catchment area:

- After dropping off the patient at the CT lab at SU/Sahlgrenska, the ambulance should wait there for 20 minutes for a decision (yes/no) on direct return transport to the home hospital in cases where treatment with thrombolysis and/or thrombectomy is not indicated.
- Patients treated with thrombolysis and/or thrombectomy at SU/Sahlgrenska should be transported to the home hospital for stroke unit care within 24–48 hours (this also includes SU/Östra and SU/Mölndal). Care in the ambulance according to the transport record.

**Helicopter Transport:**

- As with other time-critical Priority 1 missions, it should be considered to shorten the time to examination/treatment.

**General prehospital triage guidelines**

In case of suspected stroke symptoms with unclear time of onset or onset within 24 hours, an ambulance must be dispatched with **Priority 1**.

The ambulance must have a checklist for the identification and documentation of:

- Symptom profile suggestive of stroke.
- Severity of stroke according to the **(m)NIHSS** (modified version of the National Institutes of Health Stroke Scale)
- Indications and contraindications for reperfusion treatment.

**Indications and Contraindications for Stroke Alert**

**Indications**

- Age ≥16 years. And a) or b) or c):

a) (m)NIHSS ≥1 and symptom onset time such that thrombolysis can be initiated <4.5 hours.

b) (m)NIHSS ≥3 and symptom onset 4.5–24 hours. Thrombectomy must be able to be initiated there <24 hours after secondary transport to SU/Sahlgrenska. In case of unclear time of onset without signs that the onset occurred >24 hours, the patient is managed as if within 24 hours.

c) (m)NIHSS <3 and symptom onset 4.5–24 hours. If symptoms include aphasia, neglect, or visual field loss, the responsible stroke attending physician at the receiving hospital can be contacted for discussion as to whether the patient may still be eligible for a stroke alert.

**Contraindications**

- Comorbidity that makes the benefit of reperfusion treatment unlikely. Contact the responsible stroke attending physician at the receiving hospital for assessment.
- Decreased level of consciousness is not an impediment to reperfusion treatment if other causes of decreased consciousness have been evaluated and the primary suspicion of stroke remains. However, a patient with decreased consciousness should initially be assessed and, if necessary, stabilized in the emergency department before further management according to the stroke alert if stroke suspicion persists.

**Actions:**

- To enable rapid reperfusion treatment, Priority 1 for ambulance transports should be applied throughout the stroke care chain concerning reperfusion.
- Prioritize departure (rapid transport).
- Ascertain and note the time of symptom onset.
- Alert the receiving healthcare unit.
- Intravenous access (green peripheral IV catheter), preferably two on the left side. If transport to SU is sought, aim for three on the left side; if not possible, two on the left (thrombectomy) and one on the right (thrombolysis) side. This action must not delay departure.
- Monitor vital signs such as oxygen saturation, respiratory rate, pulse, blood pressure, temperature, blood glucose, and level of consciousness.
- If blood glucose < 3, treat with IV glucose.
- If SpO2 < 95%, administer 1–3 L of oxygen via nasal cannula.
- In case of nausea, administer ondansetron 4 mg IV.

**Choice of Receiving Hospital**

Basic rule: All patients where reperfusion treatment can be initiated within 24 hours are transported to the nearest acute care hospital (either for thrombolysis and/or for evaluation for thrombectomy).

**Exceptions:**

1. Direct transport to SU/Sahlgrenska (CT lab at The Center for Imaging and Intervention, floor 0):

o Patients outside SU's catchment area but <45 minutes transport time to SU/Sahlgrenska and with (m)NIHSS ≥6 who can reach SU/Sahlgrenska <6 hours from symptom onset for thrombectomy and after consultation with the thrombolysis attending physician at SU/Sahlgrenska. Patients >45 minutes ambulance transport time to SU/Sahlgrenska and with (m)NIHSS ≥6, who cannot reach the nearest acute care hospital for thrombolysis (<4.5 hours from symptom onset), but can reach SU/Sahlgrenska <6 hours from symptom onset for thrombectomy and after consultation with the thrombolysis attending physician at SU/Sahlgrenska.

o For patients in ambulances equipped with video links outside SU's catchment area, who can reach SU/Sahlgrenska <6 hours from symptom onset, with (m)NIHSS ≥6 and after consultation with the thrombolysis attending physician weekdays 09:00-16:00; all other times including weekends, the regional reperfusion attending physician at SU.

1. Total time for transport to the first hospital and secondary transport to SU/Sahlgrenska yields a clear time saving if choosing a hospital other than the nearest hospital (mainly concerns the western parts of SkaS's catchment area):

o Patients with (m)NIHSS ≥6 within 24 hours after symptom onset with a transport time no more than 15 minutes longer to another hospital than to the nearest hospital are transported to the other hospital.

**Await Decision on Secondary Transport to SU/Sahlgrenska:**

- When dropping off patients with (m)NIHSS >6 at an acute care hospital (not SU/Sahlgrenska), the ambulance should wait for 20 minutes for a decision (yes/no) regarding the need for secondary transport for thrombectomy (i.e., until after assessment with CT angiography and possibly CT perfusion). This presupposes that the patient has been handed over at the CT lab and that any thrombolysis can be initiated there before the secondary transport starts. Exception if the emergency call center needs the ambulance resource for a life-threatening mission or near a shift change, where another ambulance can take over any secondary transport.

**Secondary Transport to SU/Sahlgrenska for Patients Judged to Require Thrombectomy:**

- If possible, ensure the patient has three intravenous lines (preferably on the left side). If not possible, two on the left (thrombectomy) and one on the right (thrombolysis) side. This action must not delay departure.
- If thrombolysis (inj. Tenecteplase has been given or inf. Alteplase is running):

o Check level of consciousness (RLS), mNIHSS, pulse, and blood pressure every 15 minutes.

o If blood pressure is above 185/110, contact a physician at the departure hospital for an order for antihypertensive medication. If there is a risk of blood pressure requiring treatment during transport, appropriate medication should be sent with the ambulance.

o In case of suspicion of complications (see under the heading "Complications during Thrombolysis"), contact a physician at the departure hospital. Any Alteplase infusion should be stopped.

- Transport the patient directly to Intervention 1/Neurointervention, floor 2/3 SU/S. It is recommended to use the ambulance entrance Bruna Stråket 8, SU/S.
- Alert the thrombolysis attending physician at SU/Sahlgrenska 15 minutes before arrival. Report any changes in the patient's status such as (m)NIHSS, level of consciousness (RLS), pulse, and blood pressure.
- Call the emergency orderly/porter or SU switchboard 15–20 minutes before arrival for a meeting and help with locked elevator doors, etc.
- In the ambulance before arrival at the neurointervention lab, prepare the patient for rapid management in the lab; remove the patient's shoes and prepare for removal of pants (unbutton, etc.).
- Give a brief report on the changed status inside the neurointervention lab. The report should include the blood pressure the patient had at the first check, as this is important during the thrombectomy.

**Return Transport of Patients from SU/Sahlgrenska to the Region's Other Hospitals** after acute radiological evaluation or treatment. This applies to patients who are not part of SU/Sahlgrenska's catchment area:

- After dropping off the patient at the CT lab at SU/Sahlgrenska, the ambulance should wait there for 20 minutes for a decision (yes/no) on direct return transport to the home hospital in cases where treatment with thrombolysis and/or thrombectomy is not indicated.
- Patients treated with thrombolysis and/or thrombectomy at SU/Sahlgrenska should be transported to the home hospital for stroke unit care within 24–48 hours (this also includes SU/Östra and SU/Mölndal). Care in the ambulance according to the transport record.

**Helicopter Transport:**

- As with other time-critical Priority 1 missions, it should be considered to shorten the time to examination/treatment.
